# Predictors of primary health care pharmaceutical expenditure by districts in Uganda and implications for budget setting and allocation

**DOI:** 10.1186/s12913-015-1002-1

**Published:** 2015-08-20

**Authors:** Paschal N. Mujasi, Jaume Puig-Junoy

**Affiliations:** Department of Economics and Business, Universitat Pompeu Fabra, Barcelona School of Management, Balmes 132, 08001 Barcelona, Spain; Department of Economics and Business and Centre for Research in Health and Economics (CRES), Universitat Pompeu Fabra, Ramón Trias Fargas 25-27, 08005 Barcelona, Spain

## Abstract

**Background:**

There is need for the Uganda Ministry of Health to understand predictors of primary health care pharmaceutical expenditure among districts in order to guide budget setting and to improve efficiency in allocation of the set budget among districts.

**Methods:**

Cross sectional, retrospective observational study using secondary data. The value of pharmaceuticals procured by primary health care facilities in 87 randomly selected districts for the Financial Year 2011/2012 was collected. Various specifications of the dependent variable (pharmaceutical expenditure) were used: total pharmaceutical expenditure, Per capita district pharmaceutical expenditure, pharmaceutical expenditure per district health facility and pharmaceutical expenditure per outpatient department visit. Andersen’s behaviour model of health services utilisation was used as conceptual framework to identify independent variables likely to influence health care utilisation and hence pharmaceutical expenditure. Econometric analysis was conducted to estimate parameters of various regression models.

**Results:**

All models were significant overall (*P* < 0.01), with explanatory power ranging from 51 to 82 %. The log linear model for total pharmaceutical expenditure explained about 80 % of the observed variation in total pharmaceutical expenditure (Adjusted R^2^ = 0.797) and contained the following variables: Immunisation coverage, Total outpatient department attendance, Urbanisation, Total number of government health facilities and total number of Health Centre IIs. The model based on Per capita Pharmaceutical expenditure explained about 50 % of the observed variation in per capita pharmaceutical expenditure (Adjusted R^2^ = 0.513) and was more balanced with the following variables: Outpatient per capita attendance, percentage of rural population below poverty line 2005, Male Literacy rate, Whether a district is characterised by MOH as difficult to reach or not and the Human poverty index.

**Conclusions:**

The log-linear model based on total pharmaceutical expenditure works acceptably well and can be considered useful for predicting future total pharmaceutical expenditure following observed trends. It can be used as a simple tool for rough estimation of the potential overall national primary health pharmaceutical expenditure to guide budget setting. The model based on pharmaceutical expenditure per capita is a more balanced model containing both need and enabling factor variables. These variables would be useful in allocating any set budget to districts.

## Background

Like all public sector health services in Uganda, pharmaceuticals are funded by the Government of Uganda (GoU) through taxes and donor contributions; and provided free of charge to clients at the public sector primary health care (PHC) facilities. Government funding for essential medicines is through National Medical Stores VOTE 116, an account established by the government to effectively and efficiently supply essential medicines and medical supplies to public sector health facilities. The VOTE 116 account is the main financing mechanism for essential medicines and health supplies (EMHS) for public sector health facilities in Uganda. Based on a budget that is set by the Ministry of Finance, districts are allocated funds from this account. The funds are managed by National Medical Stores (NMS), a Ministry of Health (MoH) parastatal in charge of procurement, storage and distribution of health commodities. The NMS is mandated by MOH to procure pharmaceuticals on the National Essential Medicines list for the delivery of the minimum essential health care package as defined by the MOH and to pursue cost containment measures such as procurement of generic medicines only. With approval of the District Health Administrations, health facilities routinely order pharmaceuticals from the NMS against their allocated budget [1].

Over the last five years, the GoU’s expenditure on essential medicines and health supplies has grown at a higher rate than has public health care expenditure. GoU’s expenditure on EMHS increased substantially from about 20 billion Uganda shillings (UGX) in the financial year (FY) 2009/2010 to about 85 billion UGX in the FY 2013/2014; a 325 % increase [2]. Despite the increase, current funding for medicines is still below what is required. A policy options analysis conducted in 2010 revealed that overall government funding for EMHS in Uganda was insufficient. With the exception of contraceptives and medicines for Tuberculosis all other categories of pharmaceuticals had significant funding gaps [3]. The Health Sector Strategic and Investment plan (HSSIP) 2010/11 - 2014/15 estimated that about 956 billion UGX was required to cover national needs for medicines and related health commodities (insecticide Treated Nets, reproductive health commodities, laboratory supplies, indoor residual spraying, vaccines) in FY 2012/13 [4]. However, through VOTE 116, the government of Uganda provided an allocation of 219 billion UGX for the financial year, thereby covering just over 20 % of the estimated national need [2]. This situation requires keen attention to the predictors of primary health care pharmaceutical expenditure by districts in Uganda and the way the set pharmaceutical budget is allocated to the districts.

There is need for the Uganda Ministry of Health to understand predictors of primary health care pharmaceutical expenditure among districts in Uganda in order to guide negotiations for potential national budgets and to improve efficiency in allocation of the set budget among districts based on need. Identifying the predictors of pharmaceutical expenditure in public sector health facilities in the various districts can help identify current inefficiencies in pharmaceutical expenditure and to identify factors which if modified, for example, through policy measures (e.g. training of health workers, improving staffing etc.) can help contain pharmaceutical expenditure or make current spending more efficient by ensuring that the set budget is allocated based on need.

### Study goal

For the GoU’s publicly funded EMHS program to be sustainable in the long term, it is important that potential influencers of pharmaceutical expenditures are identified, and that the degree of their influence on such expenditures is established. This study attempts to address this need. The goal of this study is thus to identify predictors of primary health care pharmaceutical expenditure by districts in Uganda, establish explanatory models of such expenditure based on the potential influence of the identified predictors and discuss implications for potential national pharmaceutical budget estimation and setting; and allocation of the set budget to the various districts by the government.

### Prior studies

Studies in Spain, England and Italy have shown socio-demographic structure, morbidity of the population, variables associated with health care utilization [5–9], location and organizational factors [10] and quality of prescriptions [8] to be associated with pharmaceutical expenditure in PHC services at the health care area level. These factors condition the demand and supply of health services and the accompanying pharmaceutical expenditure.

With the specific aim of aiding budget setting, Forster and Frost attempted to explain differences in prescribing rates and costs between family practitioner committee areas in England and Wales based on step wise Regression [9]. They concluded that 60 % of the variation in prescribing costs per patient could be explained by differences in the age/sex distribution of the population, standardized mortality rates and the supply of general practitioners (GP) per head of population. Levels of deprivation (measured by the Jarman index) were also considered but were found to be unimportant. Similar results were obtained using number of prescriptions per person rather than cost per person as the dependent variable. As part of a more general analysis of practice variation in primary care, Baker and Klein examined differences in GP prescribing rates across family health services authorities (FHSA). Using step wise regression analysis, they were able to explain 69 % of variation in prescribing rates [10]. Explanatory variables found to be important were similar to those in Forster and Frost’s study: standardized mortality ratios, the supply of GPs per capita and the proportion of the population aged over 65 years [9]. An additional variable, the number of ancillary staff per practitioner was found to be positively significant. Again, the Jarman index was not significant.

The literature carries ample evidence of attempts at identifying and evaluating the effect of determinants of pharmaceutical expenditure in various contexts. Some studies have used information on individual patient characteristics and morbidities using adjusted clinical groups (ACG) case-mix [11, 12]. Others have used aggregate sales data on hospital sales and dispensed drugs in ambulatory care, including both reimbursed expenditure and patient co-payment; adjusting for factors likely to increase or decrease future utilization and expenditure, such as patent expiries, new drugs to be launched or new guidelines from national bodies or the regional drug and therapeutics committees [13].

However such evaluations, in most cases, focus on a single category of predictors (e.g. need, demographics or policy factors) and are mainly in developed country contexts in which medicines are provided through health insurance. Additionally, most studies have used individual pharmaceutical expenditure data collected through surveys or from individual medical records. Such data is not readily available in many developing country contexts.

There remains a need for a more inclusive approach to identifying key influencers or predictors of pharmaceutical expenditures using readily available data, and for assessing their usefulness in explaining pharmaceutical expenditure and guiding pharmaceutical budget setting and allocation in developing countries like Uganda.

Using regression analysis, this paper examines various models to explain observed variations in pharmaceutical expenditure at the district level in Uganda; with recommendations for models to be used for rough national pharmaceutical budget estimation, setting and allocation to the districts.

## Methods

### Study design

This was a cross sectional, retrospective observational study using secondary administrative data.

### Conceptual framework

The study uses Andersen’s behaviour model of health services utilization as a conceptual framework. Andersen’s model was developed to study determinants of acute care health services utilisation and has since been used frequently in a variety of health services utilization studies [14]. This comprehensive model of the demand for health services was used in this study to identify independent variables likely to influence essential medicines and health supplies utilisation at the health facilities in the districts and hence expenditure. The assumption is that since they determine utilisation of health services by the population, the identified independent variables influence the generated pharmaceutical expenditure as a result of utilization of the health services.

The model posits that health services use is broadly determined by societal factors, health services system factors and individual or population factors. Individual or population factors are categorized as predisposing factors, need, and enabling factors. Predisposing factors pertain to socio-demographics and include age, sex, marital status, education, race/ethnicity, occupation; as well as a set of beliefs (e.g. attitudes toward health services, knowledge about disease, and values). Enabling factors are those that support or impede health care service use (e.g. income, type of health insurance); and also encompass family and community resources and accessibility of those resources. Need includes individuals’ perceived and evaluated functional capacity, symptoms, and general state of health. Specifically, Andersen’s model assumes that individuals’ use of services is a function of their predisposition to use services (predisposing factors), factors that support or impede use (enabling factors), as well as their need for health care (illness level). According to Andersen, patients’ illness level (representing the need factor) is considered as the major determinant of health care utilization.

The model has been widely used in a variety of healthcare utilization studies. In a RAND study, Kubrin used Andersen’s model to develop and test predictive expectations about the role of health insurance in the use of hospital and physician services [15]. Chen and Chang used the model to construct independent variables to investigate factors associated with prescription drug expenditures among children based on the medical expenditure panel survey [16]. Chern, Wan, and Begun used the model to examine the relative importance of determinants in predicting future use of dental health services among a nationally representative sample of patients with HIV [17]. Kim and Kim employed the model to estimate future demand for institutional long-term care by Koreans [18]. Galbraith, Wong, Kim, and Newacheck used Andersen’s model to select predisposing, enabling, and need variables as independent variables that could affect health care use and expenditures in a multivariate analysis [19]. Roy and Madhavan used the model to develop an explanatory model for state Medicaid per capita drug expenditures in United States [20]. Heider et al. also employed the model to analyze health care costs in the German elderly population [21]. No published research, however, was found that used this model to study pharmaceutical expenditure in Uganda.

### Variables and data collection

**Fig. 1:**
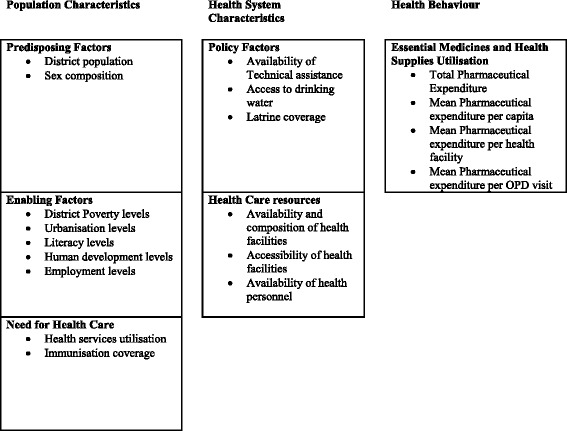
Conceptual framework. Modified Andersen’s model: Operational framework for identification of variables. Each box represents a construct which is described/measured by identified variables mentioned in the bullets. A complete list of variables their descriptions and measurement is presented in Table 1

Andersen’s model of health services utilisation was used as the operational framework for identification of variables in this study (Fig. 1). A list of the study variables, their descriptions, measurements, and sources is presented in Table 1.

**Table 1:** Description of study variables

Variable type	Variable	Description	Measurement	Data source
Predisposing factors	DISTPOP	District Population	Total projected district population,2012	UNBS
	PERCFEM	District Female Population	Percentage of Female district population, 2012	UNBS
Enabling factors	RURALPOV	Rural poverty	Percentage of rural district population below poverty line, 2005	NHS, 2005
	HDI	Human Development Index	Composite index generated from life expectancy, education attainment (adult literacy and gross enrolment) and Gross Domestic Product per capita	UDHR, 2007
	HPI	Human Poverty Index;	Index generated from measures of a long and healthy life (probability at birth of not surviving to age 40); Knowledge (adult illiteracy rate) standard of living (% of without sustainable access to an improved water source and % of children under-weight for age).	UDHR, 2007
	URBANISATION	Urbanization level	Percentage of district considered to be urban	NC, 2002
	LABOURABSRATE	Labour Absorption Rate	Percentage of population of working age (15–65) who are employed	NC 2002
	LITRATETotal	Total Literacy Rate	Percentage of the population age 15 and above who can, with understanding, read and write a short, simple statement on their everyday life.	SUPR, 2008
	LITRATEFemale	Female Literacy Rate	Percentage of the Female population age 15 and above who can, with understanding, read and write a short, simple statement on their everyday life.	SUPR, 2008
	LITRATEMale	Male Literacy Rate	Percentage of the Male population age 15 and above who can, with understanding, read and write a short, simple statement on their everyday life.	SUPR, 2008
	DISTAGE	District Age	Whether it’s a newly created district or not. = 1 if Yes; =0 if Not	MOH APR 2011/12
	DISTACCESS	District accessibility	Whether the district is characterized by MOH as hard to reach or not. =1 if Yes; =0 if Not	MOH APR 2011/12
Need for health Care	DPT3COVER	Immunisation coverage	Percentage of children fully immunized against Diphtheria, Pertusis & Tuberculosis	MOH APR 2011/12
	OPDCAPITA	Outpatient attendance	Outpatient attendance per capita	MOH APR 2011/12
Policy factors	TA	Technical assistance	Availability of donor funded Technical Assistance to the district for Pharmaceutical Management: 1 if Yes; =0 if No	SURE
	ACCESSWATER	Access to drinking water	Percentage of district population with access to Safe drinking Water	SUPR, 2008
	LATCOVERAGE	Latrine Coverage	Percentage of households with latrine	SUPR, 2008
Health care resources	HFGOVTOT	Government Health facilities	Total Number of Government Health facilities in the district (excluding hospitals)	MOH FIR, 2012
	HOSPTOT	General Hospital services	Total Number of general Hospitals, both government and private, in the district	MOH FIR, 2012
	HFNGO	Non government health facilities	Total Number of Non Government Organization (NGO) health facilities in the district	MOH FIR, 2012
	RRHAVAIL	Referral Hospital services	Availability of Regional Referral hospital in the district: Yes = 1 No = 0	MOH APR 2011/12
	PERCHCII	Health centre IIs	Percentage of government health facilities that are HC II	MOH FIR, 2012
	PERCHCIII	Health centre IIIs	Percentage of government health facilities that are HC III	MOH FIR, 2012
	PERCHCIV	Health Centre IVs	Percentage of government health facilities that are HC IV	MOH FIR, 2012
	STAFFSTRENGTH	Staff strength	Percentage of approved staff posts filled	MOH APR 2011/12
	HFACCESS	Health facility accessibility	Percentage of the district population that live within 5 km to a health facility	SUPR, 2008

*UHDR*-Uganda Human Development Report, 2007

*SUPR*-State of the Uganda Population Report; 2008

*MOH-FI*-Ministry of Health Facility Inventory Report, 2012

*NHS*-National Household Survey, 2005

*MOH ARP*-Ministry of Health Annual Performance Report, 2011/2012

*SURE*-Securing Ugandan’s Right to Essential Medicines Project Report 2011

*NC*-National Census; 2002

*UNBS*-Uganda National Bureau of Statistics

Like in many studies, a possible study approach would have been to choose just one way of expressing pharmaceutical expenditure and then go ahead to estimate the regression equation [5–7]. We however took the approach of specifying a different equation for each way of expressing pharmaceutical expenditure, just like some other studies [8, 10].

Thus, the dependent variable; pharmaceutical expenditure by all facilities in the district; was measured in several ways as below:

- Total Primary Health Care Pharmaceutical Expenditure; PHCPETotal: Value in UGX of pharmaceuticals supplied by NMS to health facilities in each district in one financial year
- Primary Health Care Pharmaceutical Expenditure per capita; PHCPECapita: Average value in UGX of pharmaceuticals supplied by NMS to health facilities in each district in one financial year per district inhabitant based on projected 2012 district population
- Primary Health Care Pharmaceutical Expenditure per outpatient visit; PHCPEVisit: Average value in UGX of pharmaceuticals supplied by NMS to health facilities in each district in one financial year per reported number of outpatient department (OPD) visits in PHC health facilities in the district during the financial year
- Primary health care Pharmaceutical expenditure per PHC health facility; PHCPEFacility: Average value in UGX of pharmaceuticals supplied by NMS to health facilities in each district in one financial year per reported number of total PHC facilities in the district

Consent from patients was not required, as this study reports expenditures at the district level not at the patient level. Data on the value of pharmaceuticals supplied by NMS to health facilities in each of the 87 randomly selected districts in Uganda for a one year period was obtained from MOH for FY 2011/2012 (July 1 2011-June 30, 2012). The data excluded pharmaceuticals supplied to district, regional and national referral hospitals since these are considered secondary and tertiary health care facilities. It also excluded data on centralized budget lines for pharmaceuticals that are mainly donor funded. These include artmesinin based combination therapies (ACTs) for Malaria; antiretrovirals (ARVs) and tuberculosis supplies; reproductive and maternal health supplies; commodities for health emergencies and vaccines for immunizations.

The independent variables were identified as:

- policy variables that capture information on interventions to improve pharmaceutical management in the district and programs aimed at improving health status of the district population
- health care resource variables that describe access to health services
- predisposing variables that describe demographic characteristics of district population
- enabling resources variables that describe factors that facilitate or impede the ability of the district population to seek health care
- need for health care variables that describe demand for health care or factors that influence health status of the district population (e.g. immunization coverage)

Data for the independent variables was collected from various government data bases and reports (Table 1).

### Study sample

A random sample of 87 districts was selected from the 112 districts that were operational in Uganda during the 2011/2012 financial year.

### Analysis methods

The unit of analysis was the district. We used IBM SPSS Statistics Version 20 to perform univariant descriptive analysis to ascertain the shape of the distribution of each variable and to discover existence of outliers. Summary statistics (maximum, minimum, mean, standard deviation) were used for this analysis. We also performed bivariant descriptive and inferential analysis to measure association between study variables and to compare means between groups of districts. For this analysis, Pearson’s coefficient and tests of equality of means were used. Finally, we performed econometric analysis using step wise multiple regression (with a stepping method criteria of probability of F-to enter of 0.05 and probability of F-to remove of 0.10) to estimate parameters of various regression models. This was done by estimation of ordinary least squares (OLS) and hypothesis tests of the value coefficients. We run both linear-linear and log-linear models for each definition of pharmaceutical expenditure in order to select the model with the best fit. Table 2 shows the variables used in the multiple linear regression analysis to determine variations in pharmaceutical expenditure among the study districts.

**Table 2:** Factors entered in multiple regression procedure to determine variations in pharmaceutical expenditure

Continuous variables	Minimum (000)	Maximum (000)	Mean (000)	Standard deviation	Correlation coefficient with			
					PHCPETotal	PHCPEcapita	PHCPEVisit	PHCPEFacility
Dependent Variable								
PHCPETotal	86476	951622	326787.04	194122.894				
PHCPECapita	0.33	2.21	1.1034	0.47168				
PHCPEVist	0.33	4.46	1.1034	0.57175				
PHCPEFacility	6974.01	50863.8	13039.4179	5382.5054				
Explanatory Variables								
Predisposing Factors								
POPTOT	54000	1723300	316959.77	217044.77	.672**	−.323**	−0.16	.520**
PERCFEM	0.4	0.55	0.506	0.01888	.251*	−0.164	−0.011	0.165
Enabling Factors								
RURALPOV	7.74 %	85.56 %	38.61 %	17.73 %	−0.118	−.318**	−.305**	−.315**
HDI	0.292	0.644	0.53572	0.060393	0.161	0.085	0.071	.260*
HPI	9.6	65.3	30.345	8.5006	−0.081	0.12	0.015	−.250*
LABOURABSRATE	16.30 %	70.70 %	52.95 %		0.008	0.034	0.062	−0.106
URBANISATION	1.10 %	100.00 %	8.19 %	11.34 %	0.211	−0.148	−0.193	.607**
LITRATETotal	0.80 %	93.70 %	65.05 %	14.63 %	.264*	0.061	0.146	.275**
LITRATEFemale	0.80 %	92.20 %	57.16 %	15.74 %	.269*	0.105	0.198	.323**
LITRATEMale	14.80 %	95.40 %	74.46 %	12.53 %	0.208	0.19	0.135	.250*
Need for Health Care								
OPDCAPITA	0	3.4	1.127	0.5194	0.181	.455**	−.460**	−0.014
DPT3COVER	0.00 %	100.00 %	82.13 %	24.28 %	−0.021	0.164	0.032	0.109
Policy Factors								
ACCESSWATER	14.60 %	97.60 %	56.75 %	16.36 %	0.058	−.296**	−0.141	0.129
LATCOVERAGE	9 %	98 %	67.96 %	18.23 %	.222*	0.101	−0.007	0.152
Health Care resources								
HFGOVTOT	7	88	26.2184	15.49375	.839**	0.179	−0.041	−0.183
HOSPTOT	0	28	1.36	3.118	.411**	−0.126	−0.189	.690**
HFNGO	0	31	7.21	6.486	.576**	−0.044	−0.059	.347**
PERCHCII	0.19	0.85	0.5731	0.15026	0.081	0.053	−0.034	−.497**
PERCHCIII	0.12	0.7	0.3594	0.13804	−0.093	−0.084	0.009	.362**
PERCHCIV	0	0.21	0.0675	0.04229	0.015	0.087	0.089	.583**
STAFFSTRENGTH	19.00 %	86.60 %	54.76 %	13.94 %	0.117	−0.142	−0.157	0.099
HFACCESS	43.70 %	96.50 %	69.85 %	11.06 %	0.193	0.133	−0.004	.266*

*Correlation is significant at the 0.05 level (2-tailed)

**Correlation is significant at the 0.01 level (2-tailed)

## Results and discussion

### Results

Before performing the regression analysis, we conducted an independent samples t-test in order to assess the null hypothesis of no difference in pharmaceutical expenditure between groups of districts for all the dichotomous variables in our data set. The results are shown in Table 3.

**Table 3:** Comparison of the means of indicators of pharmaceutical expenditure (‘000) according to levels of dichotomic variables

Availability of RRH (RRHAVAIL)	Yes (*n* = 13)	Std deviation	No (*n* = 74)	Std deviation	Means difference	t	Sig. (2 tailed)
PHCPETotal	566434.88	147749.606	284686.74	169652.114	−281748.144	−5.619	0.000
PHCPECapita	1.1587	0.35773	1.1159	0.42920	−0.04274	−0.338	0.736
PHCPEVist	0.9020	0.25030	1.1398	0.60613	0.23780	1.388	0.169
PHCPEFacility	16062.9092	10805.53931	12508.2640	3608.78774	−3554.64519	−2.247	0.027
Newly created district (DISTAGE)	Yes (*n* = 23)	Std deviation	No (*n* = 64)	Std deviation	Means difference	t	Sig. (2 tailed)
PHCPETotal	190402.76	78370.452	375800.14	200163.186	185397.383	4.312	0.000
PHCPECapita	1.1484	0.41230	1.1129	0.42244	−0.03546	−0.347	0.729
PHCPEVist	1.3383	0.86995	1.0214	0.40019	−0.31689	−2.294	0.024
PHCPEFacility	12958.6832	4150.29242	13068.4319	5790.50282	109.74865	0.083	0.934
Hard to reach district (DISTACCESS)	Yes (*n* = 18)	Std deviation	No (*n* = 69)	Std deviation	Means difference	t	Sig. (2 tailed)
PHCPETotal	281134.11	147940.892	338696.50	203694.745	57562.390	1.122	0.265
PHCPECapita	1.2578	0.52717	1.0870	0.38084	−0.17081	−1.558	0.123
PHCPEVist	1.2199	0.58504	1.0764	0.56955	−0.14350	−0.904	0.369
PHCPEFacility	11270.8536	3246.31591	13500.7825	5741.22578	2229.92887	1.579	0.118
Availability of TA for Pharmaceutical Management (TA)	Yes (*n* = 34)	Std deviation	No (*n* = 53)	Std deviation	Means difference	t	Sig. (2 tailed)
PHCPETotal	413249.53	194700.748	271320.53	173902.561	−141929.001	−3.544	.001
PHCPECapita	1.1870	0.42871	1.0808	0.40914	−0.10624	−1.160	.249
PHCPEVist	1.0605	0.48092	1.1320	0.62806	0.07155	0.563	0.575
PHCPEFacility	12533.7598	3121.21665	13363.8023	6438.73667	830.04242	0.700	0.486

The null hypothesis of no difference in total primary health care pharmaceutical expenditure among districts was rejected for districts with a regional referral hospital, newly created districts and districts with external technical assistance for pharmaceutical management (*P* < 0.05). The null hypothesis of no difference in primary health care pharmaceutical expenditure per health facility was only rejected for districts with a regional referral hospital (*P* < 0.05). The null hypothesis of no difference in primary pharmaceutical expenditure per OPD visit between districts was only rejected for newly created districts. However, the null hypothesis of no difference in primary health care pharmaceutical expenditure per capita between districts was not rejected for all the variables considered (*P* > 0.05).

We run various models (including log transformations) for each dependent variable (definition of pharmaceutical expenditure) using step wise multiple linear regression analysis. Table 4 shows the selected models (based on Adjusted R^2^) for each definition of pharmaceutical expenditure. Both linear-linear and log-linear models are presented. All the models were significant over all (*P* < 0.01). The explanatory power of the models ranged from 51 to 82 %.

**Table 4:** Multiple regression models explaining pharmaceutical expenditure

	Model 1	Model 2	Model 3	Model 4	Model 5	Model 6	Model 7	Model 8
	PHCPETotal	InPHCPETotal	PHCPEFacility	InPHCPEFacility	PHCPECapita	In. PHCPECapita	PHCPEVisit	InPHCPEVisit
Constant	43478.008	11.417*	13657.011*	9.467*	−0.500	−1.168*	3.061*	−0.372
POPTOT	0.226*							
OPDCAPITA					0.320*	0.345*	−0.580*	−0.465*
OPD TOTAL		0.00000056*						
DPT3COVER		0.003	36.566*	0.003*				
RURALPOV					−0.012*	−0.009*	−0.016*	−0.010*
HPI					0.026*	0.021*		0.020*
LABOURABSRATE			−91.918*	−0.007*			−0.014	
URBANISATION		−0.014	−122.072	−0.010				
LITERATETOT			78.085*	0.006*				0.009*
LITRATEMale					0.012			
LITRATEFemale						0.008		
DISTAGE								
HFGOVTOT	9970.501*	0.053*						
HCIITOT		−0.39*						
PERCHCII	−159186.625*		−9569.914*	−0.776*				
PERCHCIII								
PERCHCIV			31533.892*	2.467*				
DISTACCESS					0.362*	0.341*	0.461*	0.384*
N	87	87	87	87	87	87	87	87
F	99.325	64.453	15.064	15.801	18.067	15.655	13.543	21.764
R^2^	0.892	0.809	0.547	0.558	0.543	0.507	0.413	0.589
Adjusted R^2^	0.821	0.797	0.510	0.523	0.513	0.475	0.382	0.562
Significance	0.000	0.000	0.000	0.000	0.000	0.000	0.000	0.000

*(*p* < 0.01)

It is important to note that in this case, we cannot compare the different models based on the adjusted R^2^ since the models had different definitions of primary health care expenditure (dependent variable). Instead the adjusted R^2^ should only be interpreted as the variation in the specified primary health expenditure (e.g. total pharmaceutical expenditure or per capita pharmaceutical expenditure) that is explained by the variables in the model.

The model (Model 1) based on total primary health care pharmaceutical expenditure (PHCPETotal) explained about 82 % of the observed variation is total primary health care expenditure among the study districts (Adjusted R^2^ = 0.821). Apart from the constant, all the predictor variables in this model were found to be significant (*P* < 0.01). They include one predisposing factors variable (POPTOT) and three health care resources variables related to the number (HFGOVTOT) and composition of health facilities in the district (PERC HC II and PERCHC IV). Apart from percentage of Health Centre IIs (PERCHCII) which has a negative coefficient, all the other variables have a positive coefficient indicating that an increase in these variables leads to an increase in total pharmaceutical expenditure. For example, an increase in the total district population of 1,000 would leads to an increase of 226 UGX in the district’s total primary health care pharmaceutical expenditure, all other factors remaining constant. Likewise, an increase in the total number of government health facilities by one would lead to an increase of UGX 9970.501 in the district’s total primary health care pharmaceutical expenditure, all other factors remaining constant.

The log-linear model for Total Pharmaceutical expenditure explains about 80 % of the variation in the logarithm of total pharmaceutical expenditure among the study districts (Adjusted R^2^ = 0.79). Unlike the linear model which contains only pre-disposing and health care resource variables, the log-linear model contains two need for health care variables (DPT3COVER and OPDTOTAL), one enabling variable (URBANISATION) and two health care resources variables (HFGOVTOT and HCIITOT) and hence can be considered a more balanced model. Apart from the variable related to immunisation coverage (DPT3COVER) and urbanisation (URBANISATION) all other variables in the model are significant (*p* < 0.01) The Urbanisation variable and Total number of Health centre IIs variable have a negative coefficient indicating that an increase in these variables results in a decrease in observed pharmaceutical variable. For example a 1 % increase in the percentage of the district considered to be urban would lead to a decrease of 1 % decrease in the observed total pharmaceutical expenditure all other factors remaining constant. All other variables in the model had a positive coefficient indicating that an increase in these variables results in an increase in the observed total primary health care pharmaceutical expenditure.

Model 8; the log-linear model based on primary health care pharmaceutical expenditure per OPD visit explained about 56 % of the observed variation in the logarithm of primary health care expenditure per OPD visit in the study districts (Adjusted R^2^ = 0.562). Apart from the constant, all the predictor variables were found to be significant (*p* < 0.05). They include one need for health care variable (OPDCAPITA), and four enabling factors variables (RURALPOV, HPI, DISTACCESS and LITRATETOT). The OPDCAPITA and RURALPOV variables have a negative coefficient indicating that an increase in these variables results in a reduction in the pharmaceutical expenditure per OPD visit. The other variables all have a positive coefficient indicating that an increase in these variables results in an increase in the primary health care pharmaceutical expenditure per OPD visit. For example, according to this model, a district characterised by MOH as hard to reach would have a primary health care pharmaceutical expenditure per OPD visit of UGX 0.384 higher than a district not characterised as hard to reach, all factors remaining constant.

The model based on primary health care pharmaceutical expenditure per capita (Model 5) explained about 50 % of the observed variation in per capita primary health care pharmaceutical expenditure among the study districts (Adjusted R^2^ = 0.513). The model comprised of one health care need variable (OPDCAPITA), and four enabling factors variable (RURALOV, HPI, DISTACCESS and LITERATEMale). Apart from the constant, coefficients for all the other predictor variables for this model were significant (*P* < 0.05). The constant and RURALPOV had a negative coefficient, indicating that an increase in the percentage of rural district population below poverty line leads to a decrease in primary health care pharmaceutical expenditure per capita. For example, a 1 % increase in the proportion of a district’s rural population below the poverty line would lead to a decrease in primary health care pharmaceutical expenditure per capita of UGX 0.012, all other factors remaining constant. The coefficients for all the other variables were positive indicating that an increase in these variables would lead to an increase in primary health care pharmaceutical expenditure per capita. For example, a district that is characterised by MOH as hard to reach would have a per capita pharmaceutical expenditure of UGX 0.362 higher than a district that is characterised as not hard to reach, all other factors being constant.

For primary health care pharmaceutical expenditure per health facility, the selected log-linear model (Model 4) comprised of two health care resources factor variables (PERCHCIV, PERCHCII), one need for health care factors variable (DPT3COVER) and three enabling factors variables (URBANISATION, LITRATETOT, LABOURABSORPTION ). Together, these factors explain 52 % of the observed variation in the primary health care pharmaceutical expenditure per health facility in the study districts (Adjusted R^2^ = 0.522). All predictor variables in this model and the constant were significant (*p* < 0.05). The variables PERCHCII, URBANISATION, and LABOURABSRATE had a negative coefficient indicating that an increase in these variables leads to a decrease in primary health care pharmaceutical expenditure per district health facility. The other variables had a positive coefficient indicating that an increase in these variables would lead to an increase in primary health care pharmaceutical expenditure per district health facility. For example, a 1 % increase in the district DPT3 coverage would result in a 0.3 % increase in the primary health care pharmaceutical expenditure per district health facility all other factors remaining constant.

### Discussion

This study aimed at identify predictors of primary health care pharmaceutical expenditure by districts in Uganda and to establish explanatory models of such expenditure based on the potential influence of the identified predictors. The established explanatory models would be useful for rough estimation of potential national primary health care pharmaceutical budget based on previous expenditure and to guide budget setting discussions between ministry of Finance and Ministry of Health. The models would also be useful in improving equity by allocating any set budget among the various districts by the government of Uganda based on need. Like in many studies, a possible study approach would have been to choose just one way of expressing pharmaceutical expenditure and then go ahead to estimate the regression equation [5–7]. We however took the approach of specifying a different equation for each way of expressing pharmaceutical expenditure, just like some other studies [8, 10]. This is important because whereas one model may be useful in predicting future expenditure, it may not be ideal for guiding budget allocation especially if the variables in the model are not related to need, since ideally this should be the basis for budget allocation.

The variables in the model for total primary health care pharmaceutical expenditure explain about 82 % of the observed variation in total pharmaceutical expenditure among the study districts; and hence the model can be accepted as a good fit for the data. However, this model contains only health care resources variables related to the number and composition of health facilities in the district. The log-linear model for Total Pharmaceutical expenditure whose variables explain about 80 % of the observed variation in total pharmaceutical expenditure is a more balanced model since in addition to health care resource variables, it also contains need and one enabling factor variable. This balanced model works acceptably well and would be useful for predicting total pharmaceutical expenditure.

Based on the above argument and the study data, the proposed model for predicting total district primary pharmaceutical expenditure in Uganda is shown in Table 5.

**Table 5:** Proposed model for predicting total primary health pharmaceutical expenditure by districts in Uganda

Variables	Coefficient	Standard error	Student’s t	Significance
Constant	11.417*	0.134	85.441	0.000
OPDTOTAL	0.00000056*	0.000	3.085	0.003
DPT3COVER	0.003	0.001	2.277	0.026
URBANISATION	−0.014	0.005	−2.569	0.012
HFGOVTOT	0.053*	0.007	7.677	0.003
HCIITOT	−0.39*	0.009	−4.461	0.000

Dependent Variable: InPHCPETotal *n* = 87; R^2^ = 0.809 Adjusted R^2^ = 0.797; F = 64.453 Significance =0.000

*(*p* < 0.01)

This model could be used by MOH for rough estimation of future expenditure and to guide negotiations with Ministry of Finance for budget setting.

The proposed prediction model comprises of two health care resources variables related to availability and composition of health facilities in the district. As expected, an increase in the available number of health facilities is predicted to increase health care utilisation and health expenditure, hence the positive coefficient for the variable total government health facilities in the district. The other variable relate to the breakdown of the available health facilities in terms of level of care. Health Centre IIs are the lowest level of care, with few staff and limited ability to address a wide range of health conditions. A district with a higher number of Health Centre IIs among its health facilities is expected to have a lower total pharmaceutical expenditure compared to a district with a lower number of Health Centre IIs, all other factors being constant. This explains the negative coefficient for the variable number of health centres IIs in the district. As expected, the variable related to health care utilisation, total OPD attendance has a positive coefficient; an increase in total district health care utilisation is expected to increase total pharmaceutical expenditure. The negative coefficient for urbanisation may be related to the fact that urban centres have more private health care facilities which draw away patients from public sector health facilities leading to a reduction in the observed total pharmaceutical expenditure in the public sector health facilities.

Different approaches have been used to predict Pharmaceutical expenditure and to guide budget setting, including use of morbidity data and use of diagnosis and pharmacy claims data [22–26]. The predictive model developed in this study uses historical pharmaceutical expenditure data. There are a number of problems using historical costs as a basis for budget setting. Besides the lack of guarantee that the current expenditure is efficient and the possibility of manipulation by health managers who may have the incentive to increase their current expenditure with the expectation of larger budgets in the future, for better accuracy, adjustments may need to be made for the impact of certain contextual factors not included in the model. The predicted pharmaceutical expenditure using this model could for example be refined by adjusting for factors likely to increase or decrease future utilisation and expenditure such as new drugs to be introduced in the health systems or new treatment guidelines to be introduced [26].

A key strength of this study is that uses various factors in Andersen’s health services utilisation model adapted to data in a developing country context to predict primary health pharmaceutical expenditure using readily available pharmaceutical procurement data. Most prior studies of this nature have focused on a single category of predictors (e.g. need, demographics or policy factors) and are mainly in developed country contexts that have medicines provided through health insurance [9, 17, 19, 20]. Additionally, unlike this study, most studies have used individual pharmaceutical expenditure data collected through surveys or from individual medical records [11, 12].

Findings from this study could have important applications for the government of Uganda regarding setting and allocating primary health care pharmaceutical budgets. The log-linear model based on total primary health care pharmaceutical expenditure would for example be used to predict future total district primary health care pharmaceutical expenditure based on any plans the government may have for the district (e.g. increasing the number of health facilities, upgrading facilities etc.). The predicted total district PHC pharmaceutical expenditure for all districts would then be added together to predict the potential national PHC pharmaceutical expenditure. This would then be used by MOH to negotiate and set the national PHC pharmaceutical budget with Ministry of Finance.

The log-linear model based on total primary health care pharmaceutical expenditure may however not be ideal for allocating the set national primary health pharmaceutical budget among the various districts based on the needs of the population. This is because this model relies mainly on enabling and health care resources variables (HFGOVTOT, HC II TOT, and URBANISATION).

Ideally, need should be the major determinant of health care utilisation and should guide resource allocation. Based on this argument, the model of pharmaceutical expenditure per capita (Model 5) which contains need and health care pre-disposing variables would be useful for identifying factors to be considered in allocating the nationally set PHC pharmaceutical budget among districts once received by MOH based on negotiations conducted with Ministry of Finance using the log linear model based on total pharmaceutical expenditure.

Based on this model, proposed variables to be considered in allocating the set budgets among districts include the following need and pre-disposing health variables: OPD capita attendance, percentage of rural population below poverty line 2005, Male Literacy rate, Whether a district is characterised by MOH as difficult to reach or not and the Human poverty index.

The outpatient department attendance per capita (OPDCAPITA) variable is a direct reflection of demand for health care and therefore need. The expenditure generated from this demand is geared towards meeting the expressed need. The higher the demand the higher the expenditure. This calls for a higher budget allocation. This is supported by the positive coefficient of this variable in the model.

The model includes four enabling factor variables two socioeconomic variables which are the percentage of the district rural population below the poverty line 2005 (RURALPOV) and the Human Poverty Index (HPI), Male Literacy rate and whether a district is characterised by MOH as hard to reach or not. One would expect that the higher the percentage of rural poor living below the poverty line, the higher the incident of diseases and hence the higher the observed pharmaceutical expenditure, justifying a higher budget allocation. In such a situation, one would expect the variable RURALPOV to have a positive coefficient, contrary to what is observed in this study. It is also possible that given their poverty status, the poor may not be able to access health care hence leading to low expenditure in an area where the poor are the majority [22]. Such a scenario would lead to the RURALPOV variable having a negative coefficient as observed in this study. This could however be an indication of inequity in the current allocation which needs to be investigated.

The HPI measures deprivations in four dimensions: a long and healthy life-defined by vulnerability to death at a relatively early age- as measured by the probability at birth of not surviving to age 40; knowledge- defined by exclusion from the world of reading and communications- as measured by the percentage of adults (aged 16–65) lacking functional literacy skills; a decent standard of living, as measured by the percentage of people living below the income poverty line (50 % of the median adjusted household disposable income); and social exclusion as measured by the rate of long-term unemployment (12 months or more) [27]. The closer the index is to 0, the better, indicating the absence of human poverty; while the closer it is to 100, the more deprived the population is. The selected model suggests that more deprived districts should be given a higher budget allocation since one would expect a more deprived population to have higher health needs and hence higher pharmaceutical expenditure and higher budget allocation.

One variable which does not appear in the log –linear prediction model based on the total pharmaceutical expenditure, but appears in the allocation model that is based on primary health care pharmaceutical expenditure per capita is the variable related to whether a district is considered by MOH to be a hard to reach district or not (DISTACCESS). In the allocation model (Model 5) the variable is significant and has a positive coefficient. This suggests that districts that are characterized by MOH as hard to reach have a higher expenditure and should be allocated higher primary health care pharmaceutical budgets than other districts. MOH characterizes districts as hard to reach based on geography, among other factors. Geography can play an important role in influencing both individual health status and access to health services [28]. It is plausible that being hard to reach might affect the need for pharmaceuticals; for example in an area that is geographically distant, there may be an optimal substitution towards greater pharmaceutical use, since pharmaceuticals are typically more transportable than most other types of resource. Including this variable in the proposed allocation formulae offer a means to balance geographic disparities. However, it is difficult to differentiate legitimate factors related to geography that influence need for pharmaceuticals from spurious, supplier induced disparities in expenditure [28].

### Limitations

The findings of this study could have been influenced by the study limitations. Some of the data for the explanatory variables was based on past national surveys and have not been updated. For example, the Human Poverty Index data used is based on the national survey conducted in 2007, and the rural poverty data used is for 2005. The assumption that these indicators have remained constant over the period in all districts of the country may not be entirely true. Any changes that have happened in these variables may result in either under or over estimation of the various parameters of the models due to inaccurate measurement of the variable. Also, through re-districting, many new districts have been created over the period by breaking up large districts into smaller ones. Data for new districts was missing for variables obtained from national surveys conducted before the districts were created. Gaps in data were filled by allocating the same variable value to a new district as the parent district. Whereas this was the best approach to fill gaps in the circumstances, it assumes homogeneity among all counties in the district, which may not necessarily be true.

The study did not take into account centralized pharmaceutical budget lines which cover pharmaceuticals for Malaria, HIV/AIDS, Family Planning and Tuberculosis. These “program” medicines are mainly funded by donors and more funds are used for their procurement compared to essential medicines which were considered in the study. It is estimated that 60 % of health commodity financing in Uganda is donor dependent and focused on the program commodities which account for a large portion of the total Pharmaceutical expenditure in each district [3]. Specifically, ACTs are one of the most widely prescribed medicines since Malaria is the leading cause of OPD attendance in health facilities. However, spending on ACTs was not included in the study and this may have affected the results. Also, the results of this study may be subject to omitted variable bias due to the fact that data on district disease prevalence was not included as one of the study variables. Observed differences in expenditure between districts could be explained by differences in needs caused by differences in disease burden.

The value of Pharmaceuticals procured by districts from NMS was used as proxy for Pharmaceutical expenditure. This assumes that all the Pharmaceuticals procured during the financial year were dispensed and that the facility started with no stock at the beginning of the financial year. Although high stock out rates have been reported in the public sector health facilities [29], this assumption is unlikely to be true since health facilities maintain some buffer stock for a number of commodities as per the national inventory management guidelines. Using actual dispensing/pharmacy data from health facilities would have been a better reflection of actual pharmaceutical expenditure.

The study is based on Anderson’s model for acute care and the focus is on PHC which is the lowest level of care. At the macro level however, the evolution of pharmaceutical expenditures is generally driven by the entrance of new branded products and products going off-patent (genericization of the market), ageing populations, the growing prevalence of chronic disease, the disease burden confronted with, the greater use of expensive treatments and also the regulatory environment and cost-containment strategies introduced by healthcare payers. A key assumption of our study as underpinned by Andersen’s model is that utilization of health services drives utilization of pharmaceuticals and hence expenditures. As such some of the earlier mentioned factors that drive evolution of pharmaceutical expenditure at the macro level are out of our study scope and data set. This however affects the transferability potential of the suggested prediction and allocation models.

## Conclusion

The log linear model for total PHC pharmaceutical expenditure comprising of two need for health care variables (DPT3COVER and OPDTOTAL), one enabling variable (URBANISATION) and two health care resources variables (HFGOVTOT and HCIITOT) is a balanced model and has a high explanatory power. This model would be useful for MOH in negotiating and setting the national PHC pharmaceutical budget with Ministry of Finance. Although the proposed model adequately explains total district PHC pharmaceutical expenditure, its predictive value should be established as data for model variables becomes available for subsequent years.

However, a key limitation of this model is that it relies heavily on health care resources and enabling variables according to Andersen’s model. As such it may not be ideal for budget allocation to districts once the overall national budget is set. For such an allocation, the model based on pharmaceutical expenditure per capita would be ideal since it contains more factors as identified by the modified Andersen’s model. Specifically it includes need for health factors (out patient department visit per capita) which should ideally drive resource allocation if equity is to be attained.

## Abbreviations

ACG: Adjusted clinical groups
ACTs: Artemesinin based combination therapies
ARVs: Antiretrovirals (ARVs)
EMHS: Essential medicines and health supplies
FHSA: Family health services authorities
FY: Financial year
GoU: Government of Uganda
GP: General practitioner
HSSIP: Health sector strategic and investment plan
MoH: Ministry of health
NMS: National medical stores
OLS: Ordinary least squares
OPD: Outpatient department
PHC: Primary health care
UGX: Ugandan shillings
